# Strengthening human and physical infrastructure of primary healthcare settings to deliver hypertension care in Vietnam: a mixed-methods comparison of two provinces

**DOI:** 10.1093/heapol/czaa047

**Published:** 2020-07-01

**Authors:** Lana Meiqari, Thi-Phuong-Lan Nguyen, Dirk Essink, Pamela Wright, Fedde Scheele

**Affiliations:** c1 Athena Institute for Research on Innovation and Communication in Health and Life Sciences, Faculty of Sciences, Vrije Universiteit Amsterdam, De Boelelaan 1085, 1081 HV Amsterdam, The Netherlands; c2 Department of Public Health, Institute of Tropical Medicine, Kronenburgstraat 43, 2000 Antwerpen, Belgium; c3 Department of Social Medicine, Faculty of Public Health, Thai Nguyen University of Medicine and Pharmacy, 248 Luong Ngoc Quyen Street, Thai Nguyen, Vietnam; c4 Guelph International Health Consulting, De Boelelaan 1085, 1081 HV Amsterdam, The Netherlands

**Keywords:** Delivery of health care, hypertension, primary healthcare settings, Vietnam, access to care

## Abstract

In Vietnam, the overall prevalence of hypertension (HTN) was 21%, with lower estimates for the prevalence of HTN awareness and treatment. The health systems, like other low- and middle-income countries, were designed to provide acute care for episodic conditions, rather than a chronic condition where patients need long-term care across time and disciplines. This article describes the delivery and organization of HTN care at primary healthcare (PHC) settings in both urban and rural areas at Hue Province of Central Vietnam in comparison with Thai Nguyen province in Northern Vietnam based on the infrastructure capacity and patients’ and providers’ perspectives and experiences We used mixed-methods design that included in-depth semi-structured interviews with patients and healthcare providers at purposively selected PHC facilities in two districts of each province and a modified version of the service availability and readiness assessment inventory at all PHC facilities. We found that HTN patients in both provinces can access healthcare services to diagnose, treat and control their HTN condition at the PHC level with a focus on district facilities. Health services in Hue have allowed commune health stations (CHSs) to provide routine monitoring and prescription refills for HTN patients while maintaining periodical visits to a higher level of care to monitor the stability of the disease. Such provision of care at CHSs remained restricted in Thai Nguyen. Further improvements are necessary for referral procedures, information system to allow for longitudinal follow-up across levels of care and defining a basic health insurance or benefits package, which meets patients’ preferences with a monthly timespan for prescription refills.

**Key messages**

- Access to healthcare services for hypertension (HTN) patients requires flexibility to access commune health stations at the lowest level of care for routine monitoring and prescription refills, where patients would prefer a monthly timespan for prescription refills.
- Strengthening of primary healthcare infrastructure had a positive impact by enabling the system to provide HTN care for patients at the lowest level of care, nearest to their homes.
- Further improvements are needed in terms of referral procedures, information system to allow for longitudinal follow-up across levels of care and defining a basic health insurance or benefit package.

## Introduction

The global burden of cardiovascular diseases has been increasing and contributes to 31% of all deaths each year (WHO, 2019a). Hypertension (HTN) is a serious health condition and a significant risk factor for other cardiovascular and cerebrovascular diseases (D’Agostino *et al.*, 2008). In 2008, the global prevalence of HTN in adults over 25 years of age was ∼40%, with lower proportions for those who are aware of their condition and have it treated and under control (WHO, 2019b). This prevalence is targeted for a reduction of 25% by 2025 in the World Health Organization’s (WHO) Global Action Plan for the Prevention and Control of Non-Communicable Diseases (NCDs) 2013–20 (WHO, 2013). Nearly two-thirds of people with HTN live in low- and middle-income countries (LMICs), which pose a challenge to their resource-constrained health systems (WHO, 2019b). Health systems in LMICs were designed to provide acute care for episodic conditions (Robinson and Hort, 2012), while HTN is a chronic condition for which patients need long-term care across time and disciplines (Rabkin *et al.*, 2012). Consequently, the provision of chronic care requires a radical paradigm shift in how health services are managed, delivered and funded (WHO, 2015a).

The first and most used conceptualization of designing and providing care for chronic conditions is the Chronic Care Model (Wagner, 1998), which aimed to achieve improved health outcomes through the interaction of informed and empowered patients with prepared and proactive healthcare providers within the context of the health system. Also, this model highlights patients’ needs and demands and describes their active role in managing their conditions. In 2002, the Chronic Care Model was globalized through the ‘Innovative Care for Chronic Conditions (ICCC) Framework’ (WHO, 2002), which provides a road map for transforming or updating health care to meet the needs of chronic conditions for both prevention and disease management. It emphasizes the central triad, which reflects the partnerships among informed, motivated and prepared patients and families, healthcare teams and community supporters. This partnership is influenced by three levels of the health system: patient interaction (micro), healthcare organization and community (meso) and positive policy environment (macro) levels. Besides, the framework suggests building blocks at each level ‘that can be used to create or re-design a healthcare system to more effectively manage long-term health problems’.

In Vietnam, the overall prevalence of HTN was 21% and subjects with HTN had low estimates for the prevalence of HTN awareness (43%) and treatment (33%); these three estimates were significantly lower in rural areas. A few studies showed limited accommodation for HTN medications at commune health stations (CHSs), which are the entry point of care to the health system (Van Huy *et al.*, 2018; Duong *et al.*, 2019; Meiqari *et al.*, 2019b). In 2008, the Ministry of Health established the National HTN programme, which piloted a model for HTN management at the grassroots level (Nguyen *et al.*, 2011; 2012; Lim *et al.*, 2014; Meiqari *et al.*, 2019b); although this model was scaled-up gradually, activities for HTN were implemented in less than half of the CHSs by 2014 (Meiqari *et al.*, 2019b). In general, the establishment of disease- or service-specific vertical programmes has helped to provide rapid results in weak health systems and to establish better accountability and transparent governance, especially towards international organizations and funders (Atun *et al.*, 2008). However, this strategy has led to the fragmentation of health systems, not only in service delivery but also in policies and funding mechanisms (McIntyre *et al.*, 2008). More recently, countries are encouraged to focus on integrated people-centred health services based on strong primary care as a fundamental step to provide accessible, affordable, quality and equitable healthcare services (WHO, 2015a,b). For example, Vietnam is committed to achieve universal health coverage and to strengthen primary health care (PHC) (Vietnam MoH, 2016a,b; Hoang *et al.*, 2018). However, it is not clear how the health systems in LMICs can achieve their goals while addressing the needs of patients with NCDs in general and HTN in particular, especially in the context of leveraging their existing resources.

In addition, there is scarce evidence regarding the provision of HTN care in Vietnam. We performed a systematic narrative review about the available evidence on access to HTN care and services in PHC settings in Vietnam (Meiqari *et al.*, 2019b), based on the framework on people-centred access to health care (Levesque *et al.*, 2013). The framework described access as a multidimensional concept resulting from the interaction between five dimensions of accessibility for the health system, which supplies care (namely, approachability, acceptability, availability and accommodation, affordability and appropriateness) and five corresponding abilities of populations who demand care (namely, ability to perceive, to seek, to reach, to pay and to engage). The results showed an increased interest in research and policy on the burden of HTN in Vietnam. There was no information on the acceptability of HTN health services, including the interaction between patients and health professionals. Articles reported good availability of medication, but problems in accessing them such as fragmentation and lack of consistency in prescribing medication between different levels and short timespans for dispensing medication at PHC facilities. Treatment adherence among hypertensive patients did not exceed 70%.

Furthermore, Vietnam has a decentralized health service where the provincial and district levels of the health system have a certain level of authority for decision-making and resource allocation. For example, Thừa Thiên-Huế (Hue) province in Central Vietnam received international support through investments in the infrastructure of CHSs and the development of a family medicine programme, which allowed Hue’s Provincial Department of Health to deploy a family medicine doctor in most CHSs (Proscio, 2011; Hoang, 2018). Also, Hue maintained the operation of the ‘District Health Centre’ where the District Hospital (DH) is responsible for managing curative and preventive care at the district level, while Thai Nguyen has separated entities for ‘DH’ and ‘District Preventive Medicine Centre’ that are operating independently (Meiqari *et al.*, 2019b). This research aimed to describe, qualitatively and quantitatively, the delivery and organization of HTN care at PHC settings in both urban and rural areas at Hue in comparison with another province (Thai Nguyen) based on the infrastructure capacity and patients’ and providers’ perspectives and experiences. Such evidence is essential to expand the knowledge base on the role of PHC level in the provision of HTN care and to inform policymakers of needed priorities to improve HTN care.

## Materials and methods

### Study location

The study was carried out between September 2015 and June 2016 in two provinces in Vietnam, namely: Thai Nguyen Province of the ‘Northern midlands and mountain areas region’ and Thừa Thiên-Huế (Hue) Province of the ‘North Central area and Central coastal area region’. Based on the 2015 Statistical Yearbook of Viet Nam (GSO, 2016), Thai Nguyen had higher estimates for population density (337 people/km^2^) and proportion of people living in rural areas (66%) compared with Hue (227 people/km^2^ and 51%, respectively). The poverty rate in Thai Nguyen was 9.1%, higher than the national rate of 7.0% but similar to the national rate in rural areas, 9.2%. Hue’s poverty rate was lower, 4.7%. Finally, both provinces have medical universities in their urban districts, with whom the research team collaborated in conducting the research. Other key demographic and health indicators for both provinces are available in Appendix 1. Based on three studies, there are no major differences in the prevalence of HTN between the two provinces (Ha *et al.*, 2013; Bui *et al.*, 2016; Hien *et al.*, 2018) (Appendix 1).

### Study design

The design was a convergent parallel mixed-methods study (Curry and Nunez-Smith, 2014). The qualitative strand comprised semi-structured interviews (SSIs) with patients and providers at healthcare facilities, to explore the informants’ experiences with HTN care and services focusing on PHC. The quantitative strand was a cross-sectional survey to describe the human and physical resources of the PHC facilities. Data collection for qualitative and quantitative data occurred concurrently, to enable data triangulation to corroborate results from both methods and enhance their validity. Interpretation of findings followed the model of merged integration, which uses all data sources to build a coherent picture and to ensure that ’the whole is greater than the sum of the parts’ (Curry and Nunez-Smith, 2014).

### Data collection and analysis

**Figure 1:**
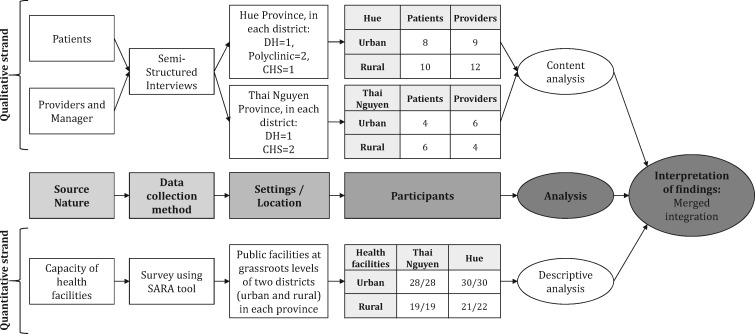
An illustration of the study design with data collection and analysis flow

Data collection and analysis for the qualitative and quantitative strands are described separately and illustrated at Figure 1; they followed the COREQ criteria for reporting qualitative research (Tong *et al.*, 2007) and the STROBE statement for reporting observational studies in epidemiology (Von Elm *et al.*, 2007).

### Qualitative strand

#### Research team

The field research team comprised the first author, two research assistants from Vrije Universiteit Amsterdam and a team of 20 research assistants and translators from the Institute for Community Health Research at Hue University of Medicine and Pharmacy and the Faculty of Public Health at Thai Nguyen University of Medicine and Pharmacy. Research assistants and translators were trained to support the data collection process.

#### Selection of settings

Each province has nine district-level administrative units, including one city and eight towns or rural districts from which two districts were selected, one urban and one rural, as seen in Table 1. We included the one city as an urban district and purposively chose one rural district for comparison with the criteria of not remote, but nearby the urban district, to decrease differences related to geographical reach and build on assumed similarities. In each district, we selected PHC facilities using a theoretical sampling with the following criteria (Emmel, 2013): (1) purposeful, to allow for exploring differences; (2) systematic, to increase the chance of uncovering similarities and differences; and (3) variational, to explore and expose variation and process. The selected PHC facilities in each district were four in Hue and three in Thai Nguyen and included: outpatient clinics at DHs, regional polyclinics (only available in Hue), and CHSs (**Table 1**).

**Table 1:** Overview of administrative units and primary healthcare facilities in Thai Nguyen and Hue provinces in Vietnam

Province	Thai Nguyen		Hue	
	Rural	Urban	Rural	Urban
	T/S	T/S	T/S	T/S
Administrative units (i.e. city or town or district)	8/1	1/1	8/1	1/1
Primary healthcare facilities				
Outpatient clinics at district hospitals	1/1	1/1	1/1	1/1
Regional polyclinics	0/NA	0/NA	2/2	2/2
Commune health stations	18/2	27/2	19/1	27/1

T/S, total number of units or facilities vs those selected for field work; NA, not applicable.

#### Sampling and participants

Each facility had two work shifts: morning and afternoon. Facilities were visited during one or more shifts. During the field visit, in-depth SSIs with patients and healthcare providers were conducted. First, HTN patients were selected at each facility using theoretical sampling with the following criteria (Emmel, 2013): (1) purposeful (i.e. non-probability), based on the patient’s characteristics (e.g. range of ages and sexes); (2) random, visits to health facilities were organized during different shifts and patients who came to the facilities during field visits were invited to participate in the study; and (3) typical case, seeking information from rich cases that is illustrative and not definitive. The number of interviews depended on the total number of patients available during field visits who agreed to participate and ranged between 1 and 3 patients per facility. Second, in-depth SSIs were conducted with 1–3 healthcare providers in each facility, including at least one doctor or assistant doctor and one nurse.

#### Data collection

Interview guides for both patients and providers were developed to capture issues related to the provision of chronic care (Wagner, 1998; WHO, 2002; Meiqari *et al.*, 2019a); they covered the following main areas: first contact, service delivery and continuity of care. Patients’ interviews started with a timeline mapping, in which patients indicated essential events in the course of their disease from diagnosis to current treatment; this exercise aimed to ‘encourage rapport building by reducing traditional hierarchies of a research interview’ and to ‘allow the participants to create a sense of direction of what they wanted to share when asked the interview questions’ (Kolar *et al.*, 2015). Interview guides were drafted in English and translated to Vietnamese and then back-translated to English in a one-on-one session during which any disagreements in translation were discussed to reach consensus and adjusted accordingly for both Vietnamese and English versions. Besides, at the end of fieldwork day, the contents of the interviews were discussed to revise interview guides. The research team sought to conduct interviews in a private room at the healthcare facility; they were conducted in Vietnamese with translation assistance into English. Interviews were audio-recorded and lasted approximately 45–60 min for patients and 60–90 min for healthcare providers. Field notes were maintained to aid data interpretation. The English version of the interview guides is available in Appendix 2.

#### Qualitative analysis

For preliminary analysis in the field, notes were taken during SSIs and then English dialogue in audio records was transcribed verbatim. At the end of the fieldwork, full audio records were transcribed verbatim in Vietnamese and then translated into English. Interview transcripts were analysed using framework analysis as described by Gale *et al.* (2013); this approach ‘sits within a broad family of analysis methods often termed thematic analysis or qualitative content analysis’ and seeks to produce descriptive conclusions clustered around themes (Gale *et al.*, 2013). This framework method follows seven stages. The first two stages (i.e. transcription and familiarization with the interviews) took place during the data collection (2016). The following five stages took place 2 years later (2018–19). First, an initial coding framework was developed through inductively coding six interviews (10% of the data). Second, based on the initial coding framework, interview guides and discussions between two team members, a working analytical framework with codes and categories was developed (available in Appendix 3). Third, the working analytical framework was applied to all interviews; this process of coding aims to create ‘a new structure for the data that is helpful to summarize/reduce the data in a way that can support answering the research questions’. The last two stages of charting and interpreting the data happened concurrently to generate themes through comparison of data codes and categories within and across cases. Consequently, two main themes were identified: (1) disease diagnosis and treatment initiation and (2) facilitators and barriers for regular monitoring and prescription refill. Coding and analysis were performed by the first author using MAXQDA 2018 (VERBI Software, 2017) and Excel Sheets.

### Quantitative strand

#### Sampling and participants

The survey targeted all public healthcare facilities located in each selected district. In each facility, data collectors approached the head or most senior health worker responsible for outpatient services available at the facility to answer questions. In addition, data collectors would validate the responses by direct observations at the facility, when possible.

#### Data collection

The survey was a modified version of the WHO’s service availability and readiness assessment (SARA) inventory (WHO, 2015c,d), which had been translated and validated by researchers from Hanoi Medical University and Hanoi University of Public Health (Duong, 2015; Van Huy *et al.*, 2018; Duong *et al.*, 2019). The SARA methodology is a standard questionnaire used to assess the availability and functioning of general services at healthcare facilities (WHO, 2010; 2015d). A data collector’s guide was developed in English and translated to Vietnamese. Data collectors were trained to administer the interviews using paper-based questionnaires; they made up to two attempts to reach the health facilities. Interviews lasted for 30–45 min. Completed surveys were entered in-field into EpiData Entry software (EpiData Association, 2013).

#### Variables

Variables used for analysis included several indicators categorized under a corresponding domain (WHO, 2010; 2015c,d); detailed definitions and measurements of indicators and domains are provided in Appendix 4. For each facility, human and physical infrastructure were assessed within three domains: (1) availability of human resources, measured as the proportion of CHSs having all five job positions (i.e. medical doctor, assistant doctor, midwife, nurse and pharmacist) as per Vietnamese national standards; (2) availability and readiness of basic infrastructure; and (3) availability and readiness of standard precautions for infection prevention. Besides, availability and readiness for HTN care activities were analysed per facility as a summary of four indicators related to guidelines and trainings on diagnosis and treatment of CVDs, basic equipment (i.e. tape measure, height ruler, adult scale, stethoscope and blood pressure measurement device) and basic medicines for HTN (i.e. ACE inhibitors, hydrochlorothiazide, beta-blockers and calcium channel blockers).

#### Statistical analysis

Data analysis included descriptive statistics, such as means and proportions of CHSs meeting specific indicators or domains. Continuous variables for readiness domain scores per facility were categorized into two groups: ≥75% and <75%. To investigate the differences between provinces and districts, *t*-test was applied to continuous variables and Chi-square or Fisher’s exact tests to categorical variables. An alpha level of .05 was used to assess statistical significance. The statistical analysis used R software (R Core Team, 2019).

### Ethics

During the consent procedures, data collectors or research assistants provided a detailed explanation to participants in Vietnamese regarding the study’s purpose, methods, risks and benefits. Participants were informed that they could refuse to answer any question or withdraw at any time and that researchers would ensure their anonymity and confidentiality at all research stages. For the qualitative strand, participants signed a written informed consent, while verbal informed consent was obtained for quantitative strand.

## Results

### Characteristics of study settings and participants

Table 2 shows the general characteristics of the 14 healthcare facilities, where data for the qualitative strand were collected. More specialist doctors were working at the DHs in Hue compared with Thai Nguyen. The number of clinical staff (including a medical doctor, assistant doctors, nurses and midwives) per 10 beds was higher in the two urban DHs compared with the rural DHs. All DHs had an outpatient clinic specified to provide care for patients with HTN or diabetes. The number of clinical staff in the urban polyclinic was double that of the rural polyclinic. However, in CHS, the number of clinical staff in Thai Nguyen at both rural and urban districts was higher than those in Hue districts, mostly due to a higher number of assistant doctors and upgraded doctors. Assistant doctors are graduates of a secondary medical school with preventive and curative duties such as diagnosis and treatment of minor cases. Some of these assistant doctors may follow additional studies to become upgraded doctors with general doctor’s duties. It is important to note that one of the CHS in the urban district of Thai Nguyen was implementing a special HTN programme.

**Table 2:** Characteristics of study settings where data for the qualitative strand were collected

	Thai Nguyen		Hue	
	Rural	Urban	Rural	Urban
Number of hospitals	1	1	1	1
Total number of beds	154	180	250	110
Total number of clinical staff	72	192	155	114
Number of clinical staff per 10 beds	5	11	6	10
Number of staff per category				
Medical doctor	25	64	32	25
General doctors	13	49	4	3
Specialist doctors	12	15	28	22
Assistant doctor	1	11	11	8
Nurse	14	49	71	56
Midwife	7	4	9	0
Pharmacists	8	10	6	12
Number of polyclinics	NA	NA	2	2
Total number of beds			30	30
Total number of clinical staff			26	63
Number of staff per category				
Medical doctor			6	12
General doctors			0	5
Specialist doctors			6	7
Assistant doctor			3	3
Nurse			7	15
Midwife			4	21
Pharmacists			2	3
Number of commune health stations	2	2	1	1
Total/average number of clinical staff per CHS	6	7	3	6
Number of staff per category				
General doctors	3	2	1	1
Assistant doctor	6	7	1	2
Nurse	3	2	1	1
Midwife	0	2	0	2
Pharmacists	1	1	0	1

NA, not applicable.

In the qualitative strand, 58 in-depth interviews were transcribed and translated (29 patients and 29 healthcare providers). As shown in Table 3, half of the patients were males, with a median age of 67 (range: 49–91 years). The median age was higher in the rural districts compared with urban ones. Almost half the patients were retired and worked as farmers, and almost two-thirds of them had not graduated from primary school. There were three types of health insurance: voluntary; social for people living in poor regions, elderly, veterans and their families and ethnic minorities; and compulsory, offered to active and retired governmental employees. Most of the patients using social insurance were living in rural districts, while most of the patients using voluntary insurance were living in urban districts. In terms of the number of years living with HTN, those interviewed in Thai Nguyen had a newer diagnosis compared with those in Hue. In addition, there was a higher gap between rural and urban Thai Nguyen as newer diagnoses were more among rural Thai Nguyen. Almost half of the patients had co-morbidities, overall and in three districts excepting rural Thai Nguyen.

**Table 3:** Characteristics of study participants in the qualitative strand, including patients and providers

	Total	Thai Nguyen		Hue	
		Rural	Urban	Rural	Urban
Total number of patients	29	6	5	10	8
Age, median (range)	67 (49–91)	73 (56–77)	58 (53–74)	71 (56–91)	66 (49–77)
Years living with HTN, median (range)	5 (0–29)	2 (0–7)	10 (0–12)	6 (1–26)	4 (2–29)
Number and proportion of patients by, *n* (%)					
Setting					
District hospital	10 (34)	2 (33)	2 (40)	4 (40)	2 (25)
Polyclinic	8 (28)	NA	NA	4 (40)	4 (50)
CHS	11 (38)	4 (67)	3 (60)	2 (20)	2 (25)
Sex, male	15 (52)	2	3	7	3
Retired/not actively working	16 (55)	1	2	5	8
Job					
Agriculture	14 (48)	4 (67)	2 (40)	8 (80)	0
Others	14 (48)	2 (33)	3 (60)	2 (20)	7 (88)
None	1 (3)	0	0	0	1 (13)
Education					
No school	4 (14)	4 (67)	0	0	0
Went to school, did not graduate	14 (48)	1 (17)	2 (40)	9 (90)	2 (25)
Went to school and graduated	7 (24)	1 (17)	2 (40)	1 (10)	3 (38)
Followed further education	4 (14)	0	1 (20)	0	3 (38)
Health insurance					
Voluntary	10 (34)	1 (17)	2 (40)	2 (20)	5 (63)
Social	14 (48)	5 (83)	0	8 (80)	1 (13)
Compulsory	5 (17)	0	3 (60)	0	2 (25)
Years living with HTN					
≤1	5 (17)	3 (50)	1 (20)	1 (10)	0
>1 to 5	11 (38)	2 (33)	1 (20)	3 (30)	5 (63)
>5	13 (45)	1 (17)	3 (60)	6 (60)	3 (38)
Co-morbidity	14 (48)	1 (17)	3 (60)	6 (60)	4 (50)
Total number of providers	29	4	5	12	8
Age, median (range)	43 (26–56)	42 (31–45)	46 (36–52)	39 (30–50)	44 (26–56)
Number and proportion of providers by, *n* (%)					
Setting					
District hospital	11 (38)	2 (50)	2 (40)	5 (42)	2 (25)
Polyclinic	9 (31)	NA	NA	5 (42)	4 (50)
CHS	9 (31)	2 (50)	3 (60)	2 (17)	2 (25)
Sex, male	12 (41)	2 (50)	1 (20)	6 (50)	3 (38)
Education					
Specialist	8 (28)	0	0	5 (42)	3 (38)
Medical degree	3 (10)	1 (25)	1 (20)	0	1 (13)
University degree	13 (45)	3 (73)	4 (80)	3 (25)	3 (38)
Intermediate college	5 (17)	0	0	4 (33)	1 (13)
Position					
Medical doctor	11 (38)	1 (25)	1 (20)	5 (42)	4 (50)
Traditional medicine doctor	1 (3)	0	0	1 (8)	0
Assistant doctors and upgraded doctor	4 (14)	2 (50)	2 (40)	0	0
Nurse or midwife	12 (41)	1 (25)	2 (40)	5 (42)	4 (50)
Pharmacist	1 (3)	0	0	1 (8)	0

NA, not applicable.

The quantitative survey was administrated at all facilities except for one CHS in rural Hue where the head of the CHS was not available for an interview following two attempts to visit. Table 4 shows the general characteristics of the human and physical resources of CHSs in Thai Nguyen and Hue. Results show a significant difference in the proportion of CHSs with a score of ≥75% for the readiness of basic infrastructure reached a higher proportion in Hue (78%) compared with Thai Nguyen (58%).

**Table 4:** The human and physical infrastructure of CHSs in Thai Nguyen and Hue

	Thai Nguyen			Hue			*P*-value
	Total (*n* = 45)	Rural (*n* = 18)	Urban (*n* = 27)	Total (*n* = 45)	Rural (*n* = 18)	Urban (*n* = 27)	
Availability of human resources for health							
Average number of health staff in a CHS, mean (SD)	7 (1.3)	7 (1.6)	6 (1.0)	7 (3.1)	7 (3.3)	7 (3.0)	0.43
Number and proportion of CHSs with at least one, *n* (%)							
1. Medical doctor	42 (93)	18 (100)	24 (89)	42 (93)	18 (100)	24 (89)	1
General doctor	40 (89)	18 (100)	22 (82)	33 (73)	16 (89)	17 (63)	0.06
Specialist doctor	**5 (11)**	0 (0)	5 (19)	**14 (31)**	4 (22)	10 (37)	**0.02**
2. Assistant doctor (of any kind)	45 (100)	18 (100)	27 (100)	45 (100)	18 (100)	27 (100)	NA
3. Nurse (of any kind)	**41 (91)**	16 (89)	25 (93)	**31 (69)**	10 (56)	21 (78)	**0.02**
4. Midwife (of any kind)	**19 (42)**	6 (33)	13 (48)	**44 (98)**	17 (94)	27 (100)	**<0,001**
5. Pharmacists (of any kind)	**17 (38)**	8 (44)	9 (33)	**30 (67)**	12 (67)	18 (67)	**0.01**
Number and proportion of CHSs with all above 5 positions	**4 (9)**	3 (17)	1 (4)	**19 (42)**	6 (33)	13 (48)	**<0,001**
Readiness of basic infrastructure, *n* (%)							
Number and proportion of CHSs with a score of ≥75	**26 (58)**	9 (50)	17 (63)	**35 (78)**	16 (89)	19 (70)	**0.04**
Readiness of standard precautions for infection prevention, *n* (%)							
Number and proportion of CHSs with a score of ≥75	38 (84)	13 (72)	25 (93)	44 (98)	17 (94)	27 (100)	0.06

NA, not applicable. Bold indicates a significant difference based on the p-value.

### HTN diagnosis and treatment initiation

**Figure 2:**
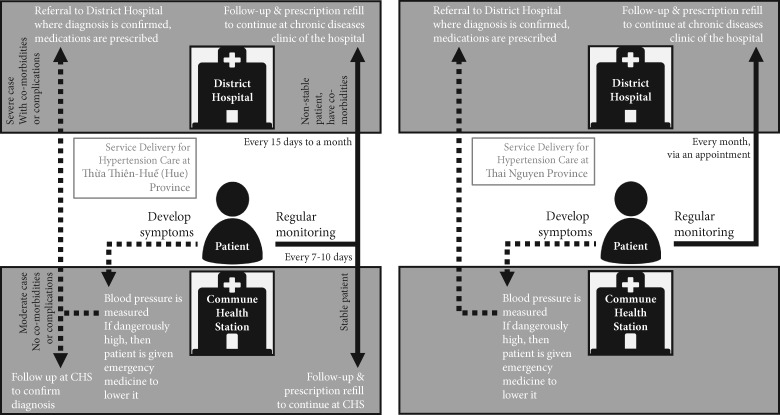
An illustration of the procedures for diagnosis and treatment initiation and regular monitoring and prescription refill for hypertension patients in Thai Nguyen and H

Figure 2 illustrates the procedures for diagnosis and treatment initiation in Thai Nguyen and Hue. Both patients and healthcare providers said that patients usually got diagnosed with HTN after noticing symptoms, mainly headaches, dizziness and fatigue. Only four patients (three in Hue and one in Thai Nguyen) discovered their HTN status accidentally through a screening project or during preparation for a surgical operation. Few patients and providers in Hue mentioned that people who are aware of the dangers of HTN have the chance to check their blood pressure at several facilities for free, e.g. at charity clinics or private pharmacies and drug stores.

*I had a headache, so I went to the pharmacy, and they measured my blood pressure. Then they said I was old and I should buy medications, after that, I should come to the health facility* (male, 68 years, polyclinic in Hue).

All patients interviewed had a health insurance subscription. Patients living in poor communes had social health insurance subsidized by the government, and formal employees had compulsory insurance through their governmental employers. Other patients reported signing up for voluntary insurance to use the public healthcare sector; a few of them started buying their health insurance after being diagnosed with HTN or diabetes. Holding a health insurance subscription became a requirement to access the public healthcare sector in January 2016. Those without insurance subscriptions still have access to public healthcare facilities, but to pay the full cost out-of-pocket. The patients’ health insurance is registered at a specific healthcare facility, depending on several factors, such as the patient’s insurance type, residency, health condition or preferences. According to the insurance policy, the patient’s entry point must be the healthcare facility where their insurance is registered; providers there can give a referral letter for higher-level facilities. Patients can go directly to higher levels of care without a referral letter, but self-referred patients bypassing the lower levels are liable for a co-payment of 30–60% of the total cost.

Symptomatic patients would first seek care at the healthcare facility where their insurance was registered; this could be a hospital’s outpatient clinic, a polyclinic or a CHS. For patients who first seek care at the lowest level (i.e. CHS), those with dangerously high blood pressure will be given emergency medications to lower their blood pressure as per guidelines and direct referral to a higher-level hospital. Patients with moderately high blood pressure will be given lifestyle advice and asked to return if the symptoms continued, to be referred to the higher-level facility. In Thai Nguyen, doctors agreed that all patients must get their final diagnosis at the hospital. In Hue, patients may be asked to return to the CHS for follow-up measurement and assessment before being referred to a DH. For patients with controlled blood pressure with or without medications, and with no additional symptoms or complaints, the healthcare provider at the CHS can confirm the HTN diagnosis and initiate the procedures for regular monitoring and prescriptions. Each CHS in both provinces had an average of 6–7 healthcare staff. However, only 9% of CHSs in Thai Nguyen reached the benchmark of having all five positions compared with 42% in Hue (*P*-value <0.001). When looking at each category, 93% of all CHSs in each province had at least one medical doctor and all CHSs had at least one assistant doctor (Table 4).

*Of course, no doctors give a diagnosis that the patients have hypertension immediately when they measure patients’ blood pressure. That is why health services require that patients must have inpatient treatment and have hospital checkout before being included in a chronic program. This rule helps to exclude other hypertension pathologies. All patients having a hypertension book must have hospital checkout because they are examined thoroughly by a doctor* (medical doctor, DH in Thai Nguyen).

*Most patents are detected at the commune health station. Then if they want to confirm their situation, they will go to a higher level [healthcare facility]* (medical doctor, DH in Hue).

Generally, most interviewed patients at both Hue and Thai Nguyen received a final diagnosis at the hospital either through a referral from CHS or by visiting the emergency department. In both provinces, two-thirds of patients interviewed reported that they started regular treatment directly after diagnosis. Only one patient in each province reported never getting regular treatment for HTN. Three patients in Thai Nguyen reported having had an earlier high blood pressure measurement without getting long-term medications for a few years, until they got rather severe symptoms, including a stroke. Also, since the CHS is not allowed to provide diagnosis and start patients’ treatment, those who do not follow the referral process have lower chances of getting both a final diagnosis and a treatment initiation directly after diagnosis. In Hue, two patients did not give a reason for not initiating treatment, while another two patients reported that they only started treatment after signing up for health insurance.

*“I came here [CHS] for a check-up, then I knew I had hypertension, so I bought the medication here. After a time, everyone said I should visit the district hospital to get an insurance notebook [and save money]. However, when I visited there, they told me to stay for a week, so I refused. I came back here to get the medication.” Question: “Why did you disagree with staying at the hospital for a week?” Answer: “Because I had too much work to do at home.”* (female, 71 years, CHS in Thai Nguyen).

*About five years ago, one day… I came home from the field; my whole body went red, and I could not breathe… I went to the CHS. My blood pressure was 190. It has been five years. I go to the commune health station every ten days to measure blood pressure and be given medications* (male, 60 years, CHS in Hue).

As per insurance guidelines, patients diagnosed with HTN will be referred to the higher-level facility where they buy a patient-retained booklet to store their HTN information, which is used for regular monitoring and prescription refills. In addition, every 3 months, HTN patients must be referred to a higher-level facility for blood tests and further diagnostic investigation. Providers use the HTN-specific patient-held booklet to record the patient’s test results, blood pressure measurements, and medication type and dosage. The booklet contains 28 pages; the first page is for patient’s name and demographic information, the second and third pages are intended to record lab results for one time only, the fourth page is for the first prescription and the following 24 pages have spaces to record blood pressure measurements and medication prescriptions, one visit per page. Patients used the same booklet for 4 months to 1 year, depending on the frequency of their clinic visits.

Finally, patients may choose to go to a private doctor or hospital. A few patients who reported following a referral or seeking care at a higher-level or a private facility had or discovered co-morbidity.

*Three years ago, I was exhausted from working too hard, so I went to the CHS, and they said I had hypertension. A few days later, I went to the private hospital for a general check-up, and they said I had hypertension and diabetes* (female, 58 years, DH in Hue).

### Facilitators and barriers for regular monitoring and prescriptions refill

A comparison of the key factors associated with HTN patients’ seeking and reaching regular monitoring and prescriptions refills in both Thai Nguyen and Hue is shown in Table 5; a few differences can be seen in the practices at both provinces.

**Table 5:** Comparison of facilitators and barriers associated with hypertension patients’ seeking and reaching of their regular monitoring and prescriptions refill at both Thai Nguyen and Hue

	Thai Nguyen	Hue	Both governorates
1. Location and timespan of regular monitoring and prescriptions refill	− HTN patients can get their daily medications from DH’s outpatient HTN and DM clinic only	+ Patients can go to either DH’s outpatient HTN and DM clinic or polyclinics or CHSs	− Patients who prefer not to go to DH buy their medications from the pharmacy store of CHSs (out-of-pocket payments) or private pharmacies
	+ Prescription refill’s timespan at DH every 28–30 days	+ Prescription refill’s timespan at DH every 28–30 days (also at polyclinics)	
	− Prescription refill at CHS is possible in few selected ones implementing a special programme with 28–30 days timespan	− Prescription refill’s timespan at CHS every 7–10 days	
2. Prescription refill’s appointment	+ Based on an appointment	− Visits are patient-initiated when the medication has run out	− Lack of recall systems or capacity to follow-up on patients who do not show up
3. Proximity from the healthcare facility		+ CHS and polyclinics are closer to the patient's residence	− DH is difficult to reach
4. Availability of adequate resources	− Equipment for diagnostic services is limited at CHSs	− Although the availability of diagnostic equipment is higher than Thai Nguyen, the services are underutilized	− HTN medication types on Health Insurance list is limited within CHSs, and more diverse in DHs
			− The stock of HTN medications at CHS is not enough; patients reported changes in their prescribed medications due to lack of supply
			− higher-level facilities (especially central and provincial hospitals) may prescribe newer generations of medications that are not available at lower levels of care, making it harder for patients to get their medications from the facilities closer to them
5. Waiting time	− Patients experience longer waiting time for their regular visit at DH	+ CHSs are perceived to have shorter waiting times compared with longer ones at DH	
6. Health Insurance Coverage	– Prescription refill at DH only	+ Prescription refill at DH or polyclinic or CHS	+ Every 3 months, patients are referred to diagnostic services at DH to have blood tests and further investigations
7. Information and records management			+ At DH, patients buy a hypertension-specific patient-held booklet to record medical history and to transfer it between different facilities
			− Information kept in the booklet (e.g. test results, BP measurements and medication type and dosage) is described as mostly incomplete

BP, blood pressure; DM, diabetes mellitus.

#### Location and timespan of regular monitoring and prescriptions refill

In Thai Nguyen, patients can only get their regular monitoring and prescription refills at the hospital level, based on a monthly scheduled appointment. In the urban district, one of the CHSs is included in a special programme where patients can also be monitored monthly. However, patients who were interviewed at all other CHSs either got their medications from the provincial or DH or bought them privately, while one patient was not on regular treatment.

*We give the patient an appointment to come back every month. If the patients get any sudden problem or a raised blood pressure, they can come back whenever they want for treatment* (female, 45 years, upgraded doctor, CHS in Thai Nguyen).

In Hue, different levels of care could provide HTN medications for patients. The timespan of regular visits was as short as every 10 days at CHSs and up to 20 or 30 days at polyclinics and DHs.

*When the hypertension patient comes, I treat them by measuring their blood pressure, giving them the prescription with medications for 10 or 15 days until the next follow-up visit* (female, 54 years, medical doctor, CHS in Hue).

Regarding the timespan of prescription, providers had to stick to the rules of the health insurance policy. Most patients who could come to the facility monthly found it convenient. However, some patients were asked to return more often, and they would have preferred to get medication for more extended periods. Some bought their medication at the pharmacy to extend the time between visits to the facility or did not take medication for some days before going back to the healthcare facility.

*Every month, I come [to the hospital] to get [my hypertension and diabetes] medications. The medicines at the pharmacy are quite expensive. I only buy medicines at the pharmacy for some days when [my medications] come to an end. Mainly, I set my mind on coming here, to the hospital* (female, 56 years, DH in Hue).

#### Scheduled appointments

In Thai Nguyen, patients who received their HTN care and medications through public healthcare facilities, either at hospitals or designated CHSs, have a planned interaction based on a monthly appointment.

*I go to [Hospital A] for an appointment to get [HTN] medications monthly, every 28 days. I have no trouble remembering the schedule because [the doctor] writes the appointment down in my booklet* (female, 60 years, CHS in Thai Nguyen).

In Hue, no scheduled appointments were planned either at hospitals or polyclinics or CHSs, but patients were asked and expected to return to the healthcare facility when running out of medication. In other words, HTN patients in Hue are responsible for the timeliness of their care. In both settings, providers can tell if the patient missed their regular visit from their medical record or the personal booklet. In that case, no follow-up is performed. However, the provider may ask patients about their refill or adherence to medications when (if) they come later.

#### Distance to the healthcare facility

Both patients and providers narrated that patients with stable conditions prefer to go to healthcare facilities closer to their residence. While this might be possible in Hue, only DHs in Thai Nguyen can prescribe HTN medications regularly under health insurance. District-level facilities were more challenging to reach for some patients, especially in rural areas.

*Question: “Why do you come today?”; Answer: “I come here to take the drug under the health insurance policy, but now they say there is no drug here. If I knew that I would buy at the pharmacy near my house. [The health insurance staff] said I have health insurance, so I come to take medication. I come here to receive the drug, but they said I must go to the district hospital to receive. It is too far for me. I always buy the drug at the pharmacy.” Question: “Where would you like to receive drugs if you had a choice?” Answer: “Here [CHS], because it is near my home.”* (female, 74 years, CHS in Thai Nguyen).

#### Availability of adequate resources

The data on CHS’s readiness to provide HTN care (Table 6) show a significant difference in the proportion of CHSs with a score of ≥75% between Hue (82%) and Thai Nguyen (51%). No significant differences were noticed between rural and urban districts in both provinces. In terms of human resources, most CHS reported having at least one healthcare provider having training on HTN prevention and control within the last 2 years. The proportion of CHS reporting access to guidelines for cardiovascular diseases (including HTN) was significantly higher in Hue (87%) than in Thai Nguyen (69%); the difference was similar for training (52% vs 38%, respectively) but was not significant.

**Table 6:** CHSs’ Readiness for hypertension activities

	Thai Nguyen			Hue			*P*-value
	Total (*n* = 45)	Rural (*n* = 18)	Urban (*n* = 27)	Total (*n* = 45)	Rural (*n* = 18)	Urban (*n* = 27)	
Hypertension							
Number and proportion of CHSs with each of the following indicator fulfilled, *n* (%)							
1. Guidelines	**31 (69)**	11 (61)	20 (74)	**39 (87)**	16 (89)	23 (85)	**0.04**
2. Trainings	15 (38)	6 (38)	9 (38)	23 (52)	8 (44)	15 (58)	0.17
3. Basic equipment	31 (69)	10 (56)	21 (78)	37 (82)	16 (89)	21 (78)	0.14
Blood pressure measurement device	44 (98)	17 (94)	27 (100)	41 (91)	17 (94)	24 (89)	0.36
Stethoscope	43 (96)	16 (89)	27 (100)	41 (91)	17 (94)	24 (89)	0.68
4. Basic medicines for hypertension	**36 (80)**	13 (72)	23 (85)	**44 (98)**	18 (100)	26 (96)	**0.02**
Calcium channel blockers	**30 (67)**	13 (72)	17 (63)	**44 (98)**	18 (100)	26 (96)	**<0,001**
ACE inhibitors	26 (58)	**7 (39)**	**19 (70)**	22 (49)	**5 (28)**	**17 (63)**	0.40
Hydrochlorothiazide	12 (27)	7 (39)	5 (19)	14 (31)	**13 (72)**	**1 (4)**	0.64
Beta-blockers	2 (4)	2 (11)	0 (0)	3 (7)	1 (6)	2 (7)	0.66
Readiness of hypertension activities, *n* (%)							
Number and proportion of CHSs with a score of ≥75	**23 (51)**	10 (56)	12 (44)	**37 (82)**	15 (83)	22 (82)	**<0,01**
Others							
Number and proportion of CHSs with each of the following indicator fulfilled, *n* (%)							
1. Protein Urine	**11 (24)**	5 (28)	6 (22)	**27 (60)**	**16 (89)**	**11 (41)**	**<0,001**

Bold indicates a significant difference based on the p-value.

Both providers and patients described a lack of medical equipment and medications, especially at commune level in both Thai Nguyen and Hue. In terms of availability and readiness of basic diagnostic services, urine tests were available in CHS in Hue, but not in Thai Nguyen. However, providers did not report on their use during the interviews. The proportion of CHS where staff reported the availability of basic medicines for HTN was significantly higher in Hue (98%) than in Thai Nguyen (80%). Providers interviewed at both rural and urban healthcare facilities reported a lack of sufficient medicines to provide adequate care to HTN patients, especially concerning types of medications. Moreover, providers at lower levels recognized their limited access to HTN medications, especially compared with higher-level facilities. These gaps in the availability of different types of basic equipment and medications were listed as primary reasons for self-referral to higher levels or to seeking care in the private sector, for patients who have the means to do either.

*For some patients, if they develop side-effects after using the medications from our list, they will go to a private doctor to get prescriptions to buy other types of medicines* (female, 52 years, medical doctor, Polyclinic in Hue).

*If the patient comes back to the CHS [after visiting the Central Hospital], we provide the medicine if we have it. Sometimes, [our] medicine is different from that given by the hospital. Here, the [types of hypertension] medications are not as diverse as available in the hospital* (male, 48 years, medical doctor, CHS in Hue).

Some patients in both provinces reported changes in their HTN medications or the need to visit the healthcare facility several times for refills as a resulted of limited supplies according to their providers. If the patient’s condition worsens or they are diagnosed with a co-morbidity, usually diabetes, they must go to a district or higher-level hospital.

#### Patient waiting times

Most interviewees reported longer waiting times at district-level facilities. DHs in Thai Nguyen reported higher workloads at daily outpatient clinic visits compared with Hue, where patients had the options to visit polyclinics and CHSs for HTN follow-up and treatment. As a consequence, providers had a short consultation time for each patient.

*Because here [at the CHS] there are few patients, they [the patients] do not have to wait too much. In the hospital, there are too many patients, so they have to wait for a long time* (female, 26 years, nurse, CHS in Hue).

#### Insurance package

Affordability and financial restrictions were not a notable concern for the patients, even for those who bought their HTN medications from drug stores and private pharmacies. Only two female patients from Hue talked about having had problems accessing HC services before they started to buy insurance.

*25 years ago, I went to the hospital in the city and found that I have hypertension. However, I did not take medications at that time; just drank some leaf teas…. I was poor at that time, going to hospital they advised me to take medication…. In 2006, I was told that hypertension patients could receive health insurance at the district hospital. I went there. Now it has been ten years, I come here once a month, sometimes every ten days or half a month. I regularly take medicine* (female, 67 years, DH in Hue).

HTN patients who visit any healthcare facility for their regular monitoring and prescription refills are entitled to referral to a higher-level facility for blood tests and further investigations once in 3 months.

#### Information and records management

To receive medications covered by health insurance, patients must bring their booklet to consultations. In other words, patients are responsible for ensuring the availability of relevant medical documents each time they visit the healthcare centre. Hospitals also have either electronic or paper-based patient records where the patient’s data are stored and can be accessed by healthcare providers. At CHSs, electronic documents with lists of HTN patients may be kept, but they are only used to report medication use and prescriptions to the district level, for health insurance purposes. Through these records, it is hard to identify the numbers of patients diagnosed, sought care, retained in care and had good control of their blood pressure.

Consequently, the transfer and sharing of a patient’s medical history between providers or healthcare facilities was also the patient’s responsibility. Providers described that, although referral letters and discharge documents are usually attached to the patient-help booklet, they are mostly incomplete. In such cases, providers have no established routines for communication for further information transfer and sharing.

#### The active role of district health centres in supporting CHSs

Given the integration of preventive and curative services at the District Health Centres in Hue, which is located at DHs, there was a routine interaction and communication between the district-level facilities and CHSs regarding resource allocations and procedures, such as medications and training, to deliver HTN care to the patients in the district at the lowest level of care. However, in Thai Nguyen, the DH focused on their role in providing direct care to HTN patients while most communications with the CHSs took place through the preventive services at the District Preventive Medicine Centre.

## Discussion

This study has shown that HTN patients in both Thai Nguyen and Hue provinces can access healthcare services to be diagnosed and treated and to control their HTN at the PHC level or the grassroots level with a focus on district facilities. Although the healthcare system in both provinces follows the same policy regarding service delivery and insurance coverage, a few differences were revealed in the implementation.

In 2018, WHO has published the HEARTS technical package for cardiovascular disease management in PHC (WHO, 2016; 2018) to provide an example of a service delivery model that define clinical and preventive services needed at primary level, in addition to the necessary structure to link to higher levels through DH and with the community through community health workers. This package comprises six elements: healthy lifestyle (H), evidence-based treatment protocols (E), access to essential medicines and technology (A), risk-based management (R), team care and task-sharing (T) and systems or monitoring (S). The HEARTS package defines the role of PHC in risk screening, assessment and management including blood pressure measurement for early detection and treatment, integrated algorithms and standardized management protocols for the management of high blood pressure, specified intervals for follow-up and re-evaluation and criteria for referral to higher levels of care. The DHs are expected to provide specialist treatment including the initial review of high-risk patients and all secondary prevention cases, review of complex cases referred from the outpatient health clinic and supervision to medical clinics in PHC. Also, the package identifies the needs of the system for support with governance, medicine supply, robust training for health workers and an expanded health information system for longitudinal follow-up. Our findings show that health services in Hue have allowed CHSs to provide routine monitoring and prescription refills for HTN patients while maintaining periodical visits to a higher level of care—in this case, the DHs—to monitor the stability of the disease. Such provision of care at CHSs remained restricted in Thai Nguyen. At the DH, patients in Thai Nguyen were supported by scheduling a regular appointment for regular monitoring and prescription refills.

Further general findings in these two provinces are consistent with evidence collected in other parts of Vietnam (Kien *et al.*, 2018) and from other LMICs, regarding care for chronic conditions in PHC settings. In particular, similarity was found in the elements associated with health systems: distance to health facility, clinical information systems, availability of essential medicines, diagnostics and trained personnel at decentralized levels of healthcare and mechanisms for coordination between public, private and alternative providers of healthcare (Lall *et al.*, 2018). All of these are also described in the strategies of the WHO Framework on integrated people-centred health services (WHO, 2015a).

One crucial step forward would be to define a basic health insurance or benefits package, which covers both preventive and disease management services for HTN at PHC level (WHO, 2016) and allows for medication dispensaries within an acceptable timespan, especially at the lowest level of care. The package should expand the types of medications available at the lowest level of care and develop treatment protocols that match the availability of resources and medications at each level of care. It should also design an educational package to inform patients about their disease throughout their care and treatment. Furthermore, there is an opportunity for experience and knowledge sharing between these two provinces or contexts in one country, where Hue province provided insights into the importance of a PHC focus and an extended timespan for repeat visits. At the same time, Thai Nguyen demonstrated a best practice by building the patient–provider relationship through an appointment system for repeat visits.

Looking at the health system, the described accessibility of health services for HTN patients at primary level in these two provinces has achieved one aspect of person-focused care and clinical integration of HTN care at the micro-level of the health system, based on the Rainbow Model of Integrated Care (Valentijn *et al.*, 2013). However, the lack of coordination across levels and sectors including an information system to allow for longitudinal follow-up across levels of care and to focus on patient outcomes is challenging clinical integration at the micro-level and organizational and professional integration at the meso-level of the health system.

Another challenge for integration and PHC is the predominance of hospital-based curative care models, which also increases the cost of service provision and the inequality between urban and rural services; this challenge is evident across health systems in both developed and developing countries (Fox, 1997), including Vietnam (Võ and Löfgren, 2019). Although DHs in Hue played a role in directing negotiations among key stakeholders to advocate for the role of CHSs, facilities at the district level require further empowerment of hospitals to coordinate PHC with lower levels of care as a crucial step to break down the hospital-centric delivery of care (WHO, 2017). In other words, changes are needed on different levels and sectors, including community, management, resource mobilization and medical education (Fox, 1997). One example is task redistribution that needs to consider the different activities and responsibilities of the CHSs and can even go to community level through ‘Community or Village Health Workers’, especially with the growing utilization of technological and mobile applications (Vedanthan *et al.*, 2017).

Key strengths of this study are the representation of the views of both patients and healthcare providers and the diversity of methods used, which allowed a rich understanding of the research question and triangulation of information. On the other hand, selection of patients through the healthcare facilities may have led to response and information bias and limited the generalizability or transferability of the findings to the whole population, in comparison with a community-based sample that would have reached a broader sample of patients who are not seeking care or are utilizing other types of health facilities such as private doctors or pharmacies.

Further studies to evaluate the integration of HTN care and services into PHC could investigate the dynamics at the macro level of the system such as the contextual factors affecting the differences in the implementation of healthcare policies across the decentralized services, in particular, the role of Vietnam Social Security at the provincial level. In addition, it would be useful to investigate the cost-effectiveness of managing patients at DHs and CHSs. Finally, further evaluation of the meso and micro levels could benefit from analysing the evidence on the impact of HTN care and services on the quality of communications between patients and providers and patient outcomes, including clinical outcomes, adherence to treatment, empowerment towards self-management and satisfaction.

## Conclusion

In conclusion, we found that Hue Province supported HTN patients by ensuring their access to services at polyclinics and CHSs, as these CHSs had significantly better readiness in infrastructure and resources capacity to manage HTN compared with Thai Nguyen. At the DH level, patients in Thai Nguyen were supported by appointment scheduling for their regular visits. Further improvements are needed in terms of referral procedures, information system to allow for longitudinal follow-up across levels of care and defining a basic health insurance or benefits package which meets patients’ preferences with a monthly timespan for prescription refills.

## Supplementary data

Supplementary data are available at *Health Policy and Planning* online.

## Supplementary Material

czaa047_Supplementary_Data
