# Supplementary material for: Strengthening human and physical infrastructure of primary healthcare settings to deliver hypertension care in Vietnam: a mixed-methods comparison of two provinces
Source: Health Policy Plan. 2020 Jul 1;35(8):918–30. doi: 10.1093/heapol/czaa047 (PMC7553760; doi:10.1093/heapol/czaa047)
Supplement: czaa047_Supplementary_Data [file czaa047_supplementary_data.zip › czaa047-Suppl_Data/3 Appendix 1.docx]

**Table 1. Key demographic and health indicators in Thai Nguyen and Hue, Vietnam, 2015**

| Indicator | National | Thai Nguyen | Hue |
| --- | --- | --- | --- |
| Population, n | 91,713,300 | 1,190,600 | 1,140,700 |
| Area, km2 | 330966.9 | 3533.2 | 5033.2 |
| Population density, person/km2 | 277 | 337 | 227 |
| Proportion of rural population, % | 66 | 66 | 51 |
| Sex ratio of population, males per 100 females | 97.3 | 96.6 | 98.5 |
| Crude birth rate, % | 16.2 | 19.0 | 15.8 |
| Crude death rate, % | 6.8 | 8.3 | 5.8 |
| Total fertility rate, children per woman | 2.10 | 2.52 | 2.26 |
| Infant mortality rate, infant deaths per 1000 live births | 14.7 | 14.7 | 20.0 |
| Under five mortality rate, under five deaths per 1000 live births | 22.1 | 22.1 | 30.2 |
| Population growth rate, % | 1.08 | 1.48 | 0.79 |
| In-migration rate, % | 5.5 | 3.5 | 3.0 |
| Out-migration rate, % | 5.5 | 4.9 | 8.4 |
| Net-migration rate, % | 0.0 | -1.4 | -5.4 |
| Proportion of literate ≥ 15 years of age population, % | 94.9 | 98.4 | 92.6 |
| Poverty rate,% | 7.0 | 9.1 | 4.7 |
| Health establishments operating under provincial department of health |  |  |  |
| Total, n | 12,791 | 211 | 179 |
| Hospital, n (% of total) | 1,036 (8%) | 19 (9%) | 18 (10%) |
| Polyclinic, n (%) | 613 (5%) | 11 (5%) | 8 (4%) |
| Medical service unit in communes, precincts, offices, & enterprises, n (%) | 11,113 (87%) | 180 (85%) | 152 (85%) |
| Medical staff working under provincial departments of health |  |  |  |
| Total, n | 230,913 | 3,572 | 2,154 |
| Doctor, n (% of total) | 57,805 (25%) | 942 (26%) | 620 (29%) |
| Doctor assistants, n (%) | 56,544 (24%) | 700 (20%) | 440 (20%) |
| Nurse, n (%) | 88,940 (39%) | 1680 (47%) | 711 (33%) |
| Midwife, n (%) | 27,624 (12%) | 250 (7%) | 383 (18%) |
| Medical staff working under provincial departments of health per 10,000 people |  |  |  |
| Total, n | 25 | 30 | 19 |
| Doctor, n | 6 | 8 | 5 |
| Doctor assistants, n | 6 | 6 | 4 |
| Nurse, n | 1 | 14 | 6 |
| Midwife, n | 3 | 2 | 3 |
| Prevalence of hypertension based on three published articles |  |  |  |
| Ha DA, 2013^A^ [4] | NA | 23.3% | NA |
| Bui, 2016^A^ [3] | 18.2% | 16.2% | 15.7% |
| Bui, 2016^B^ [3] | 14.0% | 14.2% | 12.8% |
| Hien, 2018^B^ [5] | NA | NA | 44.8% |
| Prevalence of hypertension based on three published articles among Men/Women |  |  |  |
| Ha DA, 2013^A^ [4] | NA | 30.0%/19.0% | NA |
| Bui, 2016^A^ [3] | 22.4%/14.6% | 21.9%/11.2% | 18.6%/13.2% |
| Bui, 2016^B^ [3] | 18.5%/10.2% | 20.1%/9.1% | 16.2%/9.9% |
| Hien, 2018^B^ [5] | NA | NA | 51.3%/39.7% |

^A^ Hypertension is defined as blood pressure ≥ 140/90 mmHg and/or self-reported previous diagnosis/treatment by a health professional

^B^ Measured hypertension which is defined as blood pressure ≥ 140/90 mmHg

Ha DA (study population ≥ 25 years and sample size was 2,348)

Bui (study population 25 – 64 years and sample size in Thai Nguyen 2,050 and in Hue 1,866)

Hien (study population 40 – 69 years, sample size was 983)
