# Supplementary material for: Strengthening human and physical infrastructure of primary healthcare settings to deliver hypertension care in Vietnam: a mixed-methods comparison of two provinces
Source: Health Policy Plan. 2020 Jul 1;35(8):918–30. doi: 10.1093/heapol/czaa047 (PMC7553760; doi:10.1093/heapol/czaa047)
Supplement: czaa047_Supplementary_Data [file czaa047_supplementary_data.zip › czaa047-Suppl_Data/3 Appendix 2 Interview Guide_Patient.docx]

| ***General information*** | | |
| --- | --- | --- |
| Study ID: | Province/District: | Facility: |
| ***Background*** | | |
| Age Sex Profession Education | | |
| ***Introduction to timeline*** | | |
| As a start to this interview, I would like to draw a timeline with you. The timeline will help us to better picture the course of your condition and to ask questions. I also want to be sure not to leave out important events relating to the course of your condition.  As you see, this is an empty timeline. On this line we will add important events relating to your hypertension.   - We will start with information about your health:   - What major health problems have you had that lasted for three months or more? - I now have questions about the first time you discovered your condition/s:   - How did you discover it? When did you discover it? Where were you diagnosed and treated the first time? Who did the diagnosis? - What life events have affected your chronic conditions and your health status? For example,   - Change in your work or your household, children moving away, etc. - How has the diagnosis of hypertension affected your daily life? | | |
| ***Health care provided*** | | |
| 1. Since the discovery of your condition, could you describe the care for hypertension that you have been receiving?    - What (measuring blood pressure, medication, lab tests ..etc)? Who? Where? When? Why?    - Did you have any complications or hospital admissions? 2. Why and when do you visit health facilities for your general health?    - And for hypertension in particular?  - For example, do you visit health facilities for check-ups even when you don’t have a problem with your condition?  1. Who else, apart from medical staff, helps you with your condition? (such as taking decisions on the treatment or the type of health services)  - For example, spouse, children, other relatives, traditional healer, informal care. - Who’s advice do you usually follow? Why?  1. I would like to ask you about your insurance:    - Do you have insurance?      - If yes, since when do you have insurance (before or after the diagnosis of hypertension)? What type of insurance do you have now?      - If no, why don’t you have insurance?  - Where and how do you use your insurance? - What is covered by your insurance? What is not covered? (medication, visits to healthcare providers, specialist care, etc. ) - Do you have troubles paying for your health care? If yes or no, why?  1. I would like to ask you about the medication you use for hypertension?  - What medication do you use now? - When was it prescribed? When was the last time you changed your medication? - Where do you get your medication? - Have you ever stopped taking your medications? If yes/ no, why? | | |
| ***Longitudinal continuity*** | | |
| 1. Which health facility/healthcare provider do you first visit when you have any health problem or need advice? 2. In the last 6 months, how many health care providers have you you visited when you felt sick, needed advice on your health or for regular visits?  - This could be: doctors/nurses/assistant doctors/traditional medicine doctors/traditional medicine practitioners (formal and informal)/pharmacists. - When? Where, in which facilities (this could be private or public)?  1. Have you ever used traditional medicine for your hypertension?    - If yes, how often? When? Where? Why?    - If no, why? 2. If you visit different health providers or facilities:  - Why/when do you go to see each one of these health providers or facilities including traditional medicine? - What advice or medications did you get from each one of them? Who’s advice do you follow? Who’s advice do you not follow? Why? - Are the different healthcare providers aware that you are going to other healthcare providers as well?  1. In the last 6 months, which health facility did you visit the most?  - How often did you visit it? - Do you see the same healthcare provider in that facility? - How long have you been going to this same facility/same healthcare provider?   *Main provider*   1. How important is it to you to go to the same facility or healthcare provider for your hypertension? 2. What are barriers to visiting the same healthcare provider for your hypertension? | | |
| ***Coordinated care*** | | |
| *Referral system* | | |
| 1. After your first diagnosis of hypertension, has your healthcare provider referred you to another healthcare provider/facility? (for example when you had a specific new problem, did your healthcare provider advise you to see a specialist at a hospital or to go to another healthcare provider within the same facility?) 2. Within that last 6 months, has your healthcare provider referred you to another healthcare provider/facility?  - What was the reason the last time you were referred? - Could you follow up on the referral? Why? Why not?  1. What are your suggestions on how to improve the referral process? | | |
| *Information sharing* | | |
| 1. What type of documents do you have about your medical history and condition?  - How do you use these documents?  1. What type of information did the healthcare provider you were referred have about you? (patients’ records, overview over different medication) 2. Whose responsibility was it to explain your condition to the healthcare provider you were referred to? 3. What works well in the sharing of patients’ records? 4. How could the sharing of patients’ records be improved? | | |
| ***Doctor-patient relationship*** | | |
| I am now going to ask questions regarding your relationship with the healthcare provider(s) that you visit to get the treatment and follow up for your hypertension.   1. Can you describe a typical visit to a healthcare provider that you trust? 2. What does your healthcare provider do well? 3. What could your healthcare provider do better? 4. What information does your healthcare provider have or doesn’t have about you?  - Medical history, financial situation, family situation….. etc.  1. During your visit to your healthcare provider(s), what type of information do the healthcare providers share with you? (for example management or treatment possibilities) 2. During your visit to your healthcare provider(s), do you ask questions in case something is unclear to you?  - If yes, does your healthcare provider answer your questions in ways you understand? - If no, why don’t you ask questions? - Do you ask your healthcare provider for general health advice?  1. When taking decisions about your treatment, how do your healthcare providers explain the different options available? Can you give us an example? 2. What are positive/negative aspects about your relationship to your healthcare provider(s)? | | |
| ***Patient’s own role*** | | |
| 1. Where do you get information on your condition? (e.g. healthcare providers, media, family, relatives, traditional doctors)? Which source/s do you trust the most?    - What kind of information do you get? (risk factors, information on the progress of your condition) 2. With whom do you discuss the management of your condition (e.g. family, relatives, traditional doctors, informal doctors)? 3. How does your healthcare provider encourage you to manage your own condition? Can you give an example? 4. Would you like to learn more about your condition and its management? What kind of information or management skills would you like to have? | | |
| ***Additions*** | | |
| We have talked about the long-term care you receive for your hypertension, (1) the relationship to your healthcare providers, (2) the number of healthcare providers you see and their specific role, (3) the referral process (4) and about your own role in the management of your condition.   1. How would you rate the care that you receive for your hypertension?  - What are positive aspects and negative aspects?  1. What could be improved in the care that you receive for your hypertension? How could your care be improved? 2. Would you like to add anything? | | |
| General comments: | | |
