# Supplementary material for: Strengthening human and physical infrastructure of primary healthcare settings to deliver hypertension care in Vietnam: a mixed-methods comparison of two provinces
Source: Health Policy Plan. 2020 Jul 1;35(8):918–30. doi: 10.1093/heapol/czaa047 (PMC7553760; doi:10.1093/heapol/czaa047)
Supplement: czaa047_Supplementary_Data [file czaa047_supplementary_data.zip › czaa047-Suppl_Data/3 Appendix 2 Interview Guide_Providers.docx]

| **General information** Study ID: Affiliation: |
| --- |
| **Introduction**   1. Let us start with some information on your role/position, including when you started working in this position? |
| **We are focusing on long-term care for hypertension patients**   1. Could you describe how care is organized for hypertension patients in this health facility?    1. Health providers (from who do patients receive care?)    2. Health services (what services do patients receive? For example: screening, treatment, education) 2. How many hypertension patients do you see every day? 3. Could you describe your role and responsibilities in care for hypertension patients?    1. If interviewee is a nurse -> ask if they can prescribe drugs / renew prescriptions. 4. Could you describe the patient’s role and responsibilities in caring for their hypertension? |
| **Longitudinal continuity**  ***First contact***   1. Where and how do hypertension patients usually get their first diagnosis?   ***Main healthcare provider***  *If patients go to the same facility on a regular basis and see the same health care provider, then we can say that they have a main health care provider.*   1. What type of facility/facilities do hypertension patients usually go to if they feel sick, need advice about their health, or for regular visits?    1. When patients have a usual health facility/facilities to go to, how common is it that they see the same health care provider each time?    2. How do patients choose their main healthcare provider(s)?    3. Why would patients visit/not visit their main healthcare provider(s)? 2. What type of facility/facilities do hypertension patients usually go to get their medication?    1. How do patients choose where to get their medication? 3. In your opinion, what are the advantages and disadvantages of having a main healthcare provider? 4. In the current health system, what changes are needed to encourage and enable patients to have a main healthcare provider?   ***Follow-up procedures in the health facility*** *Hypertension patients need to see a healthcare provider regularly in order to check their blood pressure and get their prescription through a follow-up visit.*   1. Could you describe how such a follow-up visit for hypertension patients is organized in this health facility? 2. When are follow-up visits scheduled? 3. How often is there follow-up with hypertension patients? 4. Who is generally responsible for follow-up visits? 5. What are the main challenges in the current follow-up system? 6. In your view, how could the current system for follow-up for hypertension patients be best improved? |
| **Patient-provider relationship**  ***Knowledge of the patient***   1. What do hypertension patients tell you during their visit? (medical & personal) 2. Could you describe the system for storing and using patient’s medical records?    1. What kind of patient information do you store?    2. How do you use these records in taking care of the patient?    3. When a patient come for a **follow-up**, how do you know about his previous visits to the clinic in terms of the blood pressure measurement, medication prescribed, test results (if available)?   ***Information sharing***   1. What do you teach hypertension patients about their condition and the treatment?    1. What type of information do you give hypertension patients?    2. In what ways do you make sure that patients understand what you tell them?    3. Where else do patients get their information/knowledge about hypertension from?   ***Partnership***   1. Could you give a description of how decisions are made regarding a patient’s hypertension care? (For example, regarding the type of medication and the follow up schedule)    1. What is the patient’s role in making decisions about the treatment?    2. How are these decisions influenced by the health insurance of the patient?    3. How prepared are patients to make decisions about the treatment?    4. How can patients be supported to make better decisions?   ***Trust***   1. How do you describe your personal relationship with your patients who come to the clinic on a regular basis? 2. How often do patients follow healthcare provider’s advices to control their high blood pressure? 3. Could you give an example of a hypertension patient that you have a good relationship with?    1. How does/did your relationship influence the care for this patient? 4. In what ways can you increase the level of trust between the patient and provider? |
| **Coordination of care**  Patients with hypertension often receive care from different healthcare providers and go to different health facilities. The following questions focus on the coordination of the care for hypertension patients between the involved healthcare providers.  ***Teamwork in the health facility***   1. How do you work together with the (other) nurse(s) / physician(s) / assistant physician(s) / pharmacist to organize care for hypertension patients?    1. How often are there meetings with the staff members in this clinic?    2. What is discussed during the meetings?   ***Referral (within levels, between levels)***   1. Could you describe how the referral system works for this health facility?    1. Where do you usually refer patients to?    2. How do you decide where to refer patients to?    3. When are patients referred (back) to you?    4. Could you give an example of when the referral system worked/did not work?    5. How is the referral of patients influenced by their health insurance? (focus on new policy)   ***Information sharing with other healthcare providers***   1. What patient information do you share with a healthcare provider when you refer a hypertension patient?    1. How does the information get to other healthcare providers? 2. How do you receive information about patients’ visits to different health facilities/healthcare providers? 3. What patient information do you receive about patients’ visits to different health facilities/healthcare providers?   ***Continuing medical education & guidelines***   1. How often do you get opportunities for continuing medical education or training?    1. About chronic care in general, and about hypertension specifically? 2. How does your health facility make use of guidelines from the government or other institutions?    1. Regarding chronic care in general, and regarding hypertension specifically? |
| **Barriers & facilitators**   1. What do you like in the current system for hypertension care? 2. What are the main challenges in the current system for hypertension care? 3. What would you like to see differently in the future? (and what should be prioritized?) |
| **Other**   1. Do you have any questions for us or would you like to add anything? |
