# Supplementary material for: Strengthening human and physical infrastructure of primary healthcare settings to deliver hypertension care in Vietnam: a mixed-methods comparison of two provinces
Source: Health Policy Plan. 2020 Jul 1;35(8):918–30. doi: 10.1093/heapol/czaa047 (PMC7553760; doi:10.1093/heapol/czaa047)
Supplement: czaa047_Supplementary_Data [file czaa047_supplementary_data.zip › czaa047-Suppl_Data/3 Appendix 3_Code Definitions_Article 4.docx]

**Appendix 1.** List and definitions of codes of the framework analysis used for this article.

| **Code/Sub-code Definition** | **Interview Guide** | |
| --- | --- | --- |
| **Provider/Info+Edu** | Provider |  |
| **Provider/Facility-related factors** |  | For example, from providers --> presence of the health professional, qualification |
| **Provider/Cont. Training** | Provider | How often do you get opportunities for continuing medical education or training? |
| **Provider/Guidelines** | Provider | How does your health facility make use of guidelines from the government or other institutions? |
| **HTN Care +** | Patient |  |
|  | Provider | What do you like in the current system for hypertension care? |
| **HTN Care -** | Provider |  |
|  | Provider | What are the main challenges in the current system for hypertension care? What would you like to see differently in the future? |
| **First Diagnosis** | Patient | Timeline |
|  | Provider | Where and how do hypertension patients usually get their first diagnosis? |
| **1st Diagnosis/Stroke** | Inductive |  |
| **1st Diagnosis/Seeking HI Center** | Inductive | HI: health insurance |
| **HTN Medications**  Including information on the accommodation of medications at the system level; additionally, to the patient’s adherence or compliance; also, this is related to issues of coordinated care in terms of having a unified treatment plan or not (When referred to different providers and facilities, unified treatment plan based on guidelines or management protocols among providers and facilities). How did the decision of getting the current medications have occurred? | Provider | What type of facility/facilities do hypertension patients usually go to get their medication? How do patients choose where to get their medication?  How do you work together with other team members at the clinic to organize care for hypertension patients? Meetings? |
|  | Patient | I would like to ask you about the medication you use for hypertension? |
| **Clinic visit description** | Inductive |  |
| **Coordinated Care/Referral Process** | Patient | Could you describe how the referral system works for this health facility? |
|  | Provider | Have you been referred? Why? Suggestions to improve referral process? |
| **Coordinated Care/Info Mgmt**  When referred to different providers and facilities,  Information management: the use and sharing of patient information over time and between providers or facilities.  This includes: record keeping and record sharing | Provider | Could you describe the system for storing and using patient’s medical records?  What kind of patient information do you store?  How do you use these records in taking care of the patient?  When a patient come for a follow-up, how do you know about his previous visits to the clinic in terms of the BP measurement, medication prescribed, test results (if available)?  What patient information do you share with a healthcare provider when you refer a hypertension patient?  How do you receive info about patient’s visits to different health facilities/providers? |
|  | Patient | Questions on information sharing/records keeping/ |
| **Utilization/Insurance/Cost** |  |  |
| **Seeking & Reaching** |  |  |
| Appointments | Inductive |  |
| Overcrowding | Inductive |  |
| Waiting Times | Inductive |  |
| Co-morbidity | Inductive |  |
| Equipment/Tests | Inductive |  |
| Travel Time/Prefer Nearest | Inductive |  |
