# Supplementary material for: Strengthening human and physical infrastructure of primary healthcare settings to deliver hypertension care in Vietnam: a mixed-methods comparison of two provinces
Source: Health Policy Plan. 2020 Jul 1;35(8):918–30. doi: 10.1093/heapol/czaa047 (PMC7553760; doi:10.1093/heapol/czaa047)
Supplement: czaa047_Supplementary_Data [file czaa047_supplementary_data.zip › czaa047-Suppl_Data/3 Appendix 4_Quan Indicator Definition.docx]

**Availability**

**1) Availability of Human Resources for Health (HRH)**

| Average number of health workers in a CHS |
| --- |
| % of CHSs having each of five types of health workers: medical doctor (general or specialist), assistant doctor, midwife (first or second-degree), nurse (first or second-degree), pharmacist (including pharmacy technician)  Specialist may include: OBGYN, paediatrics, psychology, internal medicine, surgery, family medicine, traditional medicine, ENT and Dentistry |
| % of CHSs having all five types of health workers as per Vietnamese national standards for CHSs |

**Readiness**

**1) Readiness of basic infrastructure**

| **Indicator** | **Definition** |
| --- | --- |
| 1. Power | Score = 1 when: Facility routinely has electricity from any power source during normal working hours; there has not been a break in power for more than 2 hours per day during the past 7 days; as reported by facility |
| 2. Improved water source inside OR within the ground of the facility | Score = 1 when: Improved water source includes: Piped, tubewell/borehole, protected dug well. The water source is located inside the facility or within the ground of the facility; as reported by facility |
| 3. Private Room/ Room with auditory and visual privacy for patient consultations | Score = 1 when: Private room available in main service area, where providers/clients can have a normal conversation without being overheard, and without the client being observed; as reported by facility |
| 4. Access to adequate sanitation facilities for clients | Score = 1 when: The toilet/latrine includes the following: Flush/pour flush to piped sewer system or septic tank or pit latrine |
| 5. Communication equipment | Score = 1 when: Functioning communication equipment, including: landline or cellular telephones |
| 6. Access to computer with email/internet | Score = 1 when: Facility has a functioning computer and has access to email/internet which is working on the day of the survey |
| 7. Emergency transportation | Score = 1 when: Facility has a functioning vehicle with fuel or access to a vehicle in near proximity that can be used for emergency transportation |
| Domain score per facility = mean score of indicators per facility as a percentage; i.e. (sum of indicators/7)*100 | |
| % of CHSs with scores that are (>=75 and <75) | |

**2) Readiness of standard precautions for infection prevention**

| **Indicator** | **Definition** |
| --- | --- |
| 1. Safe final disposal of sharp waste | Score = 1 when: Safe final disposal of sharps includes incineration, open burning in protected area, dump without burning in protected area, or remove offsite with protected storage; ; as observed by interviewer. If method is incineration, incinerator functioning and fuel available; as reported by facility. |
| 2. Appropriate storage of sharp waste | Score = 1 when: Observed or reported availability of sharps container (i.e. safety box) |
| 3. Safe final disposal of non-sharp medical waste | Score = 1 when: Safe final disposal of infectious wastes includes incineration, open burning in protected area, dump without burning in protected area, or remove offsite with protected storage; ; as observed by interviewer. If method is incineration, incinerator functioning and fuel available; as reported by facility. |
| 4. Appropriate storage of non-sharp medical waste | Score = 1 when: Observed availability of waste receptacle (pedal bin) with lid and plastic bin liner |
| 5. Sterilization equipment | Score = 1 when: This is either an autoclave or dry heat sterilizer with available and functioning heat sources (if relevant), in addition to use of boiling or chemicals; observed or reported availability in the facility and reported functionality. |
| 6. Environmental disinfectant | Score = 1 when: Observed or reported availability of chlorine- or alcohol-based materials used for environmental disinfection |
| 7. Single-use syringes | Score = 1 when: Observed or reported availability of single use-standard disposable or auto-disable syringes |
| 8. Soap and running water or alcohol-based rubs | Score = 1 when: Observed or reported availability |
| 9. Latex gloves | Score = 1 when: Observed or reported availability |
| 10. Guidelines for standard precautions, sterilization and waste management | Score = 1 when: Observed or reported availability |
| Domain score per facility = mean score of indicators per facility as a percentage; i.e. (sum of indicators/10)*100 | |
| % of CHSs with scores that are (>=75 and <75) | |

**3) Readiness of hypertension activities**

| **Indicator** | **Definition** |
| --- | --- |
| 1. Guidelines for diagnosis and treatment of chronic cardiovascular conditions | Score = 1 when: Observed or reported availability |
| 2. Staff trained in diagnosis and management of chronic cardiovascular conditions | Score = 1 when: At least one staff providing the service trained in diagnosis and management of chronic cardiovascular conditions in the last two years; as reported by interviewee |
| 3. Basic equipment | Score = 1 when: Observed or reported availability and reported functionality of following five items: tape measure, height ruler, adult scale, stethoscope, and Blood pressure measurement device |
| 4. Basic medicines for hypertension | Score = 1 when: Observed in pharmacy or in area where they are routinely stored, at least one with valid expiration date  These include any of four types of medications: ACE inhibitors (i.e. Captopril, Enalapril, Perindopril), hydrochlorothiazide, Beta-blockers (i.e. Atenolol, Propronolol), and calcium channel blockers (i.e. Amlodipine, Nifedipine) |
| Domain score per facility = mean score of indicators per facility as a percentage; i.e. (sum of indicators/4)*100 | |
| % of CHSs with scores that are (>=75 and <75) | |

**4) Other**

| **Indicator** | **Definition** |
| --- | --- |
| 3. Blood or urine tests | Score = 1 when: Observed or reported availability and reported functionality of protein urine test |
| % of CHSs with scores that equal 1 | |
